# Multicenter Prospective Pilot Study Identifying Thrombomodulin as a Potential Biomarker for Neurocognitive Outcomes in Immune Thrombotic Thrombocytopenic Purpura

**DOI:** 10.3390/jcm14030694

**Published:** 2025-01-22

**Authors:** Aaron B. Boothby, Michael D. Evans, Shangbin Yang, Senthil Sukumar, James G. Scott, Deirdra R. Terrell, Spero Cataland, Marshall Mazepa

**Affiliations:** 1 Division of Hematology and Oncology, University of Washington, Seattle, WA 98195, USA; 2 Clinical and Translational Science Institute, University of Minnesota, Minneapolis, MN 55455, USA; 3 Section of Hematology-Oncology, Ohio State University, Columbus, OH 43210, USA; 4 Section of Hematology-Oncology, Baylor College of Medicine, Houston, TX 77030, USA; 5 Department of Psychiatry and Behavioral Sciences, University of Oklahoma Health Sciences Center, Oklahoma City, OK 73104, USA; 6 Department of Biostatistics and Epidemiology, University of Oklahoma Health Sciences Center, Oklahoma City, OK 73104, USA; 7 Division of Hematology, Department of Medicine, Ohio State University, Columbus, OH, USA; 8 Division of Hematology, Oncology, and Transplantation, University of Minnesota, Minneapolis, MN 55455, USA

**Keywords:** thrombotic thrombocytopenic purpura, thrombotic microangiopathy, neurocognitive function

## Abstract

**Background/Objectives**: Immune thrombotic thrombocytopenic purpura (iTTP) is a rare, chronically relapsing disorder that causes life-threatening thrombotic microangiopathy. Many survivors in clinical remission show evidence of ongoing silent cerebral infarction and neurocognitive deficits. Prospective longitudinal studies of this population are needed to acquire a complete understanding of the mechanism behind this ongoing neurologic injury. We aimed to assess the feasibility of a multicenter prospective study of neuropsychological and cognitive function in iTTP survivors in remission and examine novel biomarkers. **Methods**: We aimed to enroll 50 iTTP patients across three USTMA consortium sites between 2019 and 2022 in a 24-month longitudinal study. Clinical, cognitive, and biomarker assessments, including ADAMTS13 activity, were performed. **Results**: Despite the COVID-19 pandemic, we enrolled 38 subjects, and 31 (81.6%) completed closeout evaluations at 24 months. Upon the participants’ enrollment in the study, we confirmed previous findings, including high rates of moderate to severe neurocognitive and psychiatric sequelae (anxiety, 47%; depression, 45%; and headaches, 55%). Changes in cognitive function were measurable and included decreased immediate memory and visuospatial abilities. Over this two-year study, we did not see a significant change in neurocognitive findings. There was no association between cognitive function and ADAMTS13 activity; however, we found that the level of soluble thrombomodulin (CD141) was significantly correlated with cognitive impairment. **Conclusions**: We conclude that a more extensive study is feasible, and at least 5–10 years may be required to detect trends in neurocognitive function. Soluble thrombomodulin is a promising biomarker for cognitive impairment in survivors of iTTP, and it is worthy of additional study.

## 1. Introduction

Immune thrombotic thrombocytopenic purpura (iTTP) is a rare, chronically relapsing disorder that causes life-threatening thrombotic microangiopathy (TMA). The physiology of acute episodes of iTTP was established in the 1990s: autoantibody-mediated destruction or inhibition of the von Willebrand Factor (vWF) cleaving protease ADAMTS13 leads to the formation of ultra-large vWF multimers (UL-vWF) that have high affinity for circulating platelets [1,2]. During iTTP episodes, circulating UL-vWF-platelet aggregates cause microvascular occlusion, mechanical intravascular hemolysis, and end-organ ischemia (including strokes). In the modern era, remission can be achieved with plasma exchange and immune suppression [3], leading to a decrease in mortality from a historical value of >90% to 1–20% [4,5,6,7,8]. 

With more patients surviving the acute phase, cross-sectional studies conducted to examine neurocognitive outcomes in remission have revealed concerning brain imaging findings and high rates of functional deficits, depression, headaches, and poor health-related quality of life [9,10,11,12,13,14,15,16]. In this regard, neurocognitive deficits are similar to those seen in hypertension [17], sickle cell disease [18], and multi-infarct dementia [19]. While these deficits were initially attributed to the ischemic neurologic injury during the acute TMA episodes, there is growing evidence for progressive neurologic injury occurring during periods of clinical remission. Up to 50% of survivors are found to have abnormal MRIs consistent with ongoing microvascular damage [9,14]. Despite these adverse outcomes, the current standard of care for long-term complications in iTTP patients in remission is limited to the periodic monitoring of ADAMTS13 and markers of microangiopathic hemolytic anemia [20]. Which tests may be helpful in monitoring for neurologic sequelae in iTTP patients in remission and how often they should be performed remain unclear.

We hypothesized that progressive neurologic injury in iTTP results from periods of lower ADAMTS13 activity and accumulation of UL-vWF and microvascular ischemic events, which arise and subside without a clinically apparent relapse. To explore the utility of novel biomarkers in iTTP, in consultation with neurological experts, we selected four soluble markers of endothelial injury (Syndecan-1, thrombomodulin, vascular adhesion protein-1, and E-selectin). Increased syndecan-1 and thrombomodulin have been reported to predict in-hospital mortality in the acute setting and disease recurrence in remission [21]. However, their potential role in predicting neurocognitive outcomes is not known.

Given the rarity of iTTP, and to move the field forward, a large-scale prospective multicenter study is needed to ascertain the incidence of objective neuropsychologic dysfunction during remission, define the pace of progression, allow for an estimate of the duration of an interventional trial, and assess the utility of biomarkers that may allow for the establishment of surrogate endpoints. To this end, we conducted the first prospective, multicenter, longitudinal pilot study of neurocognitive outcomes and novel biomarkers for iTTP patients in remission.

## 2. Materials and Methods

### 2.1. Study Design and Setting

We aimed to enroll 50 participants with iTTP. Between September 2019 and July 2022, patients were enrolled at one of three United States Thrombotic Microangiopathy (USTMA) consortium sites (Ohio State University, University of Oklahoma Health Sciences Center, and University of Minnesota). This study received institutional review board approval at each of the three participating sites, and patients provided written informed consent. Over two years, participants participated in study visits every three months, for a total of nine visits. Comprehensive assessments of mental health, physical health, and cognition were administered. In addition to the performance of routine clinical labs, blood samples were obtained for ADAMTS13 activity and biomarker testing.

### 2.2. Eligibility Criteria

Eligible patients were ≥18 years old and in clinical remission (ADAMTS13 remission was not required) for at least 90 days. The diagnosis of iTTP required a degree of ADAMTS13 activity ≤ 10% during an acute TMA episode.

### 2.3. Outcomes Definitions

Clinical remission was defined by attaining a platelet count ≥ 150 × 10^9^/L and LDH < 1.5 times the upper limit of normal with an ADAMTS13 activity value ≥ 20% [22]. Clinical relapse was defined as platelet count <150 × 10^9^/L and ADAMTS13 activity < 20% [22]. ADAMTS13 relapse was defined as activity dropping to <20% from prior remission [22]. 

### 2.4. Data Collection and Study Assessments

Demographics and clinical information were abstracted through medical chart review. Participants were prospectively interviewed at each study visit, and data were collected in a centralized, de-identified REDCap database. ADAMST13 testing was completed at each site via commercially available FRETS (Immucor Inc., Norcross, GA, USA) or ELISA-based (Technozym ADAMTS13 Activity, Diapharma, West Chester Township, OH, USA) assays. Biomarker assays were performed on samples from each study visit using commercially available ELISA-based assays (soluble vascular adhesion protein-1 (sVAP) (Invitrogen, Carlsbad, CA, USA), selectin (R&D Systems Inc., Minneapolis, MN, USA), syndecan (Diaclone, now Medix Biochemica, Westbrook, ME, USA), and thrombomodulin (CD141) (Abcam, Cambridge, UK). Study assessments included the Beck Anxiety Index (BAI) [23], Beck Depression Index-II (BDI) [24], Headache Impact Test (HIT-6) [25], Short Form-36v2 (SF-36, used to measure health-related quality of life) [26], and Conners Continuous Performance Test, Third Edition (CPT-3, a computer-based assessment of reaction time, attention, and processing speed) [27]. Cognition was assessed annually (upon entry, in year 1, and in year 2) using the Repeatable Battery for the Assessment of Neuropsychological Status (RBANS) [28], and the Montreal Cognitive Assessment (MoCA) [29] was substituted during the other visits. RBANS scores were compared to normative samples corrected for age, and standard scores were converted to standardized z-scores to compare them to population-normed scores and percentiles.

### 2.5. Statistical Analysis

Analyses were conducted using R version 4.2.2 (R Development Core Team) and GraphPad Prism version 10.0. Participant characteristics were summarized using frequencies and percentages or medians and interquartile ranges. The stability of biomarker levels within participants over time was measured by fitting linear mixed-effects models to calculate intraclass correlation coefficients. Associations between mean biomarker levels and mean neurocognitive testing metrics were examined using Spearman rank correlations and visualized using local regression curves. A *p*-value less than 0.05 was considered statistically significant.

## 3. Results

### 3.1. Enrollment and Study Completion

Between September 2019 and July 2022, we enrolled 38 of 50 (76%) of the planned participants (16 from Ohio State University, 13 from the University of Oklahoma Health Sciences Center, and 9 from the University of Minnesota). Enrollment was temporarily halted at all sites due to the COVID-19 pandemic. Participants were 84.2% (32/38) female, and 42.1% (16/38) self-identified as Black. The median ADAMTS13 activity value upon entry into the study was 65.7 (interquartile range: 42–97.5) (Table 1). Few participants underwent ADAMTS13 antibody testing upon enrollment; over the study period, the median antibody level was 9 (IQR 6–38). Of the 38 participants, 65% (222/342) completed the planned visits. A total of 28 participants (73.7%) completed the close-out assessment at two years, with 10 (26.3%) participants lost to follow-up.

### 3.2. Clinical Courses

Nine participants (24%) had at least one ADAMTS13 value < 20% at any study visit. Seven of these nine participants (78%) with any ADAMTS13 activity value < 20% self-identified as Black (odds ratio 6.65 (1.31–34.46, *p* = 0.0514)). One patient had persistently low ADAMTS13 activity (<20%); this was accompanied by mild thrombocytopenia (platelets < 150 × 10^9^/L) in two instances but no signs of end-organ injury that would be consistent with a relapse of iTTP (Appendix A). This patient was not treated, and their platelet count recovered without intervention. Four participants (11%) suffered an overt clinical relapse (treatment and details are given in Table 2) between study visits, and one patient was treated with caplacizumab.

### 3.3. Neurocognitive Impairment and Poor Mental Health

At enrollment, 47% (18/38) of the participants had at least moderate anxiety according to the BAI, and 45% (17/38) had at least moderate depression according to the BDI-II. HIT-6 revealed that 71% (27/38) suffered increased headaches, which were severe in 47% of cases (18/38), with a median score of 58 (IQR 47.5–63). In total, 55% of the subjects had moderate to severe headaches (21/38), while 26% (9/35) of the subjects had longer reaction times as measured by the CPT-3. Subjects frequently noted dissatisfaction with the test, severe enough to stop completion in several cases. A total of 33 subjects completed the RBANS assessment. RBANS scores were compared to normative samples corrected for age, and standard scores were converted to standardized z-scores to compare them to population-normed scores and percentiles. The RBANS scores at enrollment detected significant deviation from population norms consistent with mild cognitive impairment (median RBANS Total z-score= - 0.88, 18th percentile). Marked deviations from expected performance were observed in immediate memory (z-score = −1.10, 13th percentile) and visuospatial construction (z-score = −1.28, 10th percentile), and milder declines relative to the general population were observed in language (z-score= −0.52. 30th percentile). No significant decrease was observed in delayed memory (z-score= −0.024, 42nd percentile) and attention (z-score= −0.10, 46th percentile). Quality-of-life measures obtained using SF-36 revealed increased fatigue (with a median fatigue score of 30 and a population mean of 52.2) and pain (median, 57.5; population mean, 78.8), as well as impairments in social functioning (median, 62.5; population mean, 78.8) and general health (median, 35; population mean, 56.99) (Table 3). We analyzed the slope of the RBANS test battery scores over time for each participant; the scores did not change significantly throughout the study period.

### 3.4. Biomarker Testing

**Figure 1:**
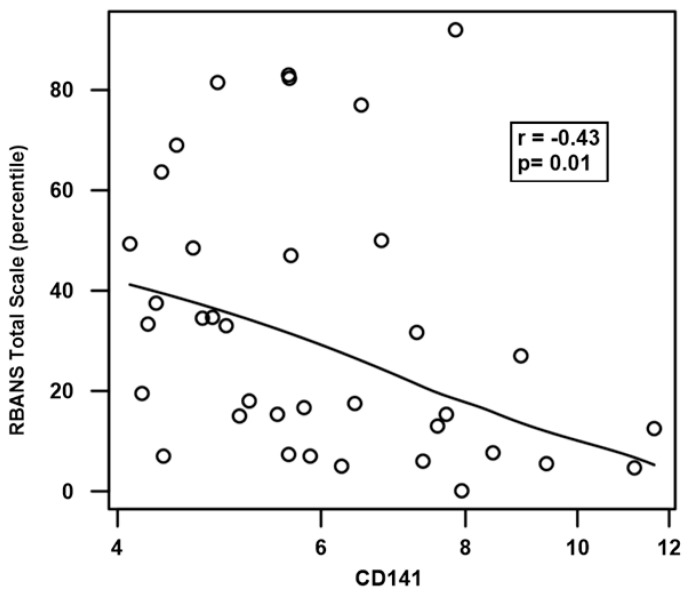
RBANS Total score percentile vs. thrombomodulin (CD141). All visits during which we measured both the RBANS and CD141 are shown. The line is a fitted local regression with Spearman correlation and *p*-value listed.

Novel biomarkers were obtained for 82% (31/38) of subjects at enrollment and in 71% of the study visits (157/222). The variability of the biomarker levels over time was quantified using the intraclass correlation coefficient and is reported in Table 3. SVAP, Selectin, Syndecan, and CD141 tended to be more stable (ICC 0.74–0.84) than ADAMTS13 (ICC 0.60) across study visits (Table 4). We observed a significant correlation between serum CD141 (thrombomodulin) and RBANS total scaled score percentile (r = −0.43, *p* = 0.01, Figure 1). This association was not seen for ADAMTS13 activity or the biomarkers collected (Syndecan-1, Vascular adhesion protein-1, E-selectin) (Appendix B).

## 4. Discussion

In this pilot study, we successfully demonstrated the feasibility of using a multicenter longitudinal study to examine neurocognitive and mental health outcomes in a cohort of patients with iTTP. Despite the COVID-19 pandemic, we enrolled >70% of our planned cohort. Our findings corroborate previous single-center reports of a high prevalence of mental health disorders, reduced physical health, and measurable cognitive deficits in survivors of iTTP [10,11,12,16]. Only a small subset of the participants suffered an overt relapse. However, we noted that the participants who self-identified as Black appeared more likely to have a low ADAMTS13 level during the study period. We found that there were no significant changes in measurements of neurocognitive function over the two-year study period, and only CD141 showed a significant correlation with neurocognitive function among the assessed biomarkers.

We were able to enroll a cohort of iTTP patients across three sites. The COVID-19 pandemic temporarily halted research at all sites and prevented the full recruitment of the planned 50 participants. The majority of these participants completed the study closeout examination at two years. The participants noted dissatisfaction with the frequency of testing and the CPT-3 test battery. We did not observe significant declines in mental, cognitive, or physical health measures over the two years of follow-up. This is not surprising given that another three-year study of iTTP survivors also reported no change in measurements of cognitive impairment [30]. However, a study of 15 iTTP patients in which cognitive function tests were repeated eight years apart showed evidence of progressive deficits compared with age-matched controls [10]. In keeping with our results, this study also found no association between cognitive function and the number of episodes of iTTP, the time since last relapse, or the ADAMTS13 activity measured (annually) during remission [10]. Given the relative stability of the biomarkers (other than ADAMTS13) and measures of neurocognitive function, we conclude that an annual assessment of these parameters will be adequate for future studies.

Our findings and those reported in prior work confirm our expectation that a more extended follow-up period would be needed (likely 5–10 years) to detect clear progression in health and cognitive function measurements. The COVID-19 pandemic impacted attrition and enrollment; however, we expect a 25% attrition rate in the subsequent study.

In our cohort, ADAMTS13 showed significant intra-individual variation over time; multiple patients had ADAMTS13 activity values < 10%; however, there were few overt clinical relapses. Most (78%) of the participants with ADAMTS13 < 20% during the study self-identified as Black. This may result from this population’s previously reported decrease in remission duration after rituximab [31]. The exact factors that result in this disparity have yet to be identified. One patient had persistently undetectable ADAMTS13 levels for the entire study period (and many years prior) without apparent sequelae. There was no association between ADAMTS13 levels and any of our assessed measures. This highlights the continued need for a better understanding of the pathophysiology of neurocognitive dysfunction in iTTP in clinical remission.

Of the other assessed biomarkers, only CD141 was associated with a neurocognitive outcome. CD141 (Thrombomodulin) is an endothelial surface transmembrane glycoprotein that regulates hemostasis, coagulation, fibrinolysis, inflammation, and angiogenesis [32,33]. While it would be intuitive to assume higher soluble thrombomodulin levels might have protective effects, trials of recombinant soluble thrombomodulin in the case of sepsis have largely been negative [34]. Further, increased soluble thrombomodulin levels reflect ongoing endothelial dysfunction and have previously been shown to be associated with relapse risk in iTTP [21,35]. In our study, higher CD141 was associated with a reduced RBANS total scale percentile, which equates to worse performance. Notably, CD141 and all the novel biomarkers assessed were stable over time in all the individuals but varied significantly between individuals. This suggests that CD141 may be a useful correlate of cognitive function in iTTP survivors, and a single baseline measure may be sufficient.

Our study was limited by inter-site heterogeneity in treatment modalities, lab testing, and a lack of access to imaging correlates. Including brain imaging in future work may provide additional insights into the utility of the assessed biomarkers and their correlation with the incidence of progressive neurologic insult in iTTP [9,14]. Another possible limitation is that more severely affected patients may have been less likely to complete the study. The COVID-19 pandemic hampered recruitment. However, the attrition rate among the participants was reasonable, and most of the testing was feasible. We conclude that a more extensive/longer-term study is feasible but should account for attrition, employ less frequent study visits, and minimize unacceptable assessments. This work will be essential to fully explore the potential clinical and therapeutic implications of these findings.

## 5. Conclusions

This pilot study establishes the feasibility and potential of using a large multicenter prospective study to examine neurocognitive and psychological outcomes for patients with iTTP in remission. We found CD141 to be a promising biomarker of neurocognitive outcomes and identified opportunities to streamline the study protocol to reduce impacts on participants.

## Figures and Tables

**Table 1:** Demographics, clinical history, and ADAMTS13 activity upon study enrollment.

Baseline Characteristics	n = 38
Female	32 (84.2%)
Black race	16 (42.1%)
ADAMTS13 activity (IQR)	65.7 (42–97.5)
Years since first iTTP episode (IQR)	13.9 (1.5–18.5)
Years since last iTTP episode (IQR)	6.3 (1–12.5)

Abbreviations: iTTP, immune thrombotic microangiopathy; IQR, interquartile range.

**Table 2:** Clinical courses of participants and treatments used during the study period.

Relapses and Treatment Agents	
ADAMTS13 activity measures < 20%	18/194
Clinical relapse *	4
Corticosteroid	5
Rituximab (clinical relapse *)	3
Rituximab (ADAMTS13 relapse *)	7
Cyclosporine	7
Other immune suppressive agents	2
Caplacizumab	1

Treatments are listed by number of courses. * Definitions per [22].

**Table 3:** Results of neurocognitive and psychological testing at enrollment.

Beck Index	Minimal	Mild	Moderate	Severe
Anxiety	29% (11/38)	24% (9/38)	29% (11/38)	18% (7/38)
Depression	37% (14/38)	18% (7/38)	29% (11/38)	16% (6/38)
**HIT-6**	**Grade 1 (normal)**	**Grade 2**	**Grade 3**	**Grade 4**
	29% (11/38)	16% (6/38)	8% (3/38)	47% (18/38)
**CPT-3**	**1–2 SD slower**			
Reaction time	26% (9/35)			
RBANS *				
**Immed. memory**	**Delayed memory**	**Visuospatial**	**Language**	**Attention**
13th (7–50)	42nd (18–58)	10th (2–39)	30th (25–53)	46th (27–67.5)
**SF-36**				
**Social function**	**Fatigue**	**Wellbeing**	**Pain**	**General health**
62.5 (40.63–83.75)	30 (20–50)	64 (48–76)	57.5 (45–86.88)	35 (21.25–60)

Abbreviations: HIT-6, headache impact test. CPT-3, continuous performance test. SD, standard deviation. Immed., immediate. * Median percentile.

**Table 4:** Biomarker stability over the study period.

Biomarker	The Intraclass Correlation Coefficient
ADAMTS13	0.60
SVAP	0.80
Selectin	0.74
Syndecan	0.84
Thrombomodulin (CD141)	0.76

The intraclass correlation coefficient (ICC) for each biomarker is shown. The ICC ranges from 0 to 1, with higher values indicating greater within-participant reliability over time.

**Figure A1:**
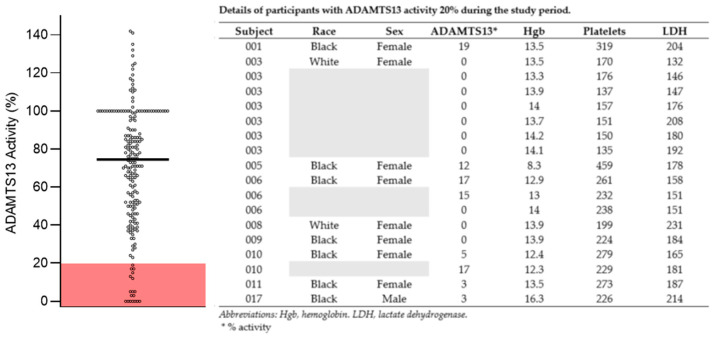
All ADAMTS13 measurements made during this study are shown; the red shaded area indicates ADAMTS13 activities < 20%. The table shows the participant-level details of these data points.

**Figure A2:**
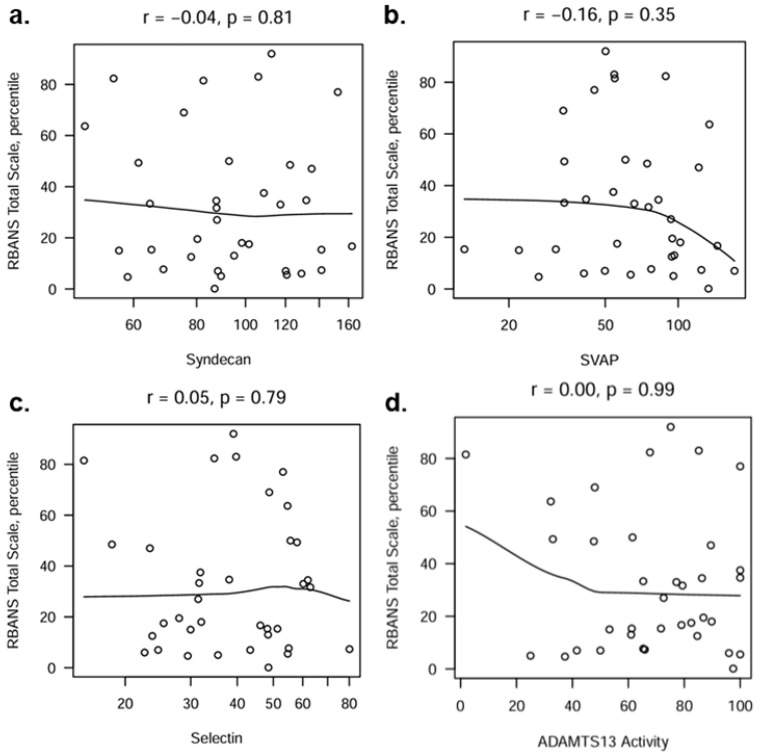
(**a**) RBANS total scale percentile vs. syndecan. (**b**) RBANS total scale percentile vs. SVAP. (**c**) RBANS total scale percentile vs. selectin. (**d**) RBANS total scale percentile vs. ADAMTS13 activity. The line is a fitted local regression, with Spearman correlation and *p*-value shown above each figure.

## Data Availability

All data reported herein are available. Raw data may are available on request from the corresponding author.
